# Urinary CXCL10 for detection of rejection and infection at 1-year posttransplant

**DOI:** 10.3389/ti.2026.17130

**Published:** 2026-10-12

**Authors:** Alain M. Häseli, Karoline Leuzinger, Matthias Diebold, Caroline Wehmeier, Joëlle Handschin, Patrizia Amico, Michael Dickenmann, Pietro E. Cippà, Helmut Hopfer, Patricia Hirt-Minkowski, Stefan Schaub

**Affiliations:** 1 Clinic for Transplantation Immunology and Nephrology, University Hospital Basel, Basel, Switzerland; 2 Clinical Virology, University Hospital Basel, Basel, Switzerland; 3 HLA-Diagnostics and Immunogenetics, Department of Laboratory Medicine, University Hospital Basel, Basel, Switzerland; 4 Institute of Pathology, University of Basel, Basel, Switzerland

**Keywords:** biomarker, BK polyomavirus, chemokine CXCL10, kidney transplantation, rejection

## Abstract

Urinary CXCL10 chemokine levels correlate with inflammatory processes in the reno-urinary tract after kidney transplantation. The aim of this study was to investigate the diagnostic characteristics of urinary CXCL10/creatinine ratio to detect major inflammatory processes (i.e., subclinical rejection, relevant JC/BK polyomavirus DNAuria, or significant leukocyturia) in 405 stable renal allograft recipients with surveillance biopsies at 1 year posttransplant. Urinary CXCL10/creatinine had a significantly higher area under the curve (AUC) than serum creatinine or proteinuria (0.786 vs. 0.499 vs. 0.636; both p < 0.0001) for detection of inflammatory processes. The highest AUC was observed for the detection of BK polyomavirus DNAuria (0.936), but it was lower for detection of subclinical rejection (0.703). At an arbitrary rule-out cutoff (CXCL10/creatinine <3 ng/mmol; 59% of all patients; negative likelihood ratio 0.36), 51% of surveillance biopsies could be safely deferred, and the risk of subclinical rejection decreased from 15.8% to 5.4%. At an arbitrary rule-in cutoff (CXCL10/creatinine >10 ng/mmol; 12% of all patients; positive likelihood ratio 12.92), absence of any inflammatory pathologies was only 2%. These data support urinary CXCL10/creatinine measurements at 1-year posttransplant in stable allografts to detect inflammatory processes and guide further diagnostics. The preliminary cutoffs require external validation.

## Graphical Abstract

**Figure:**
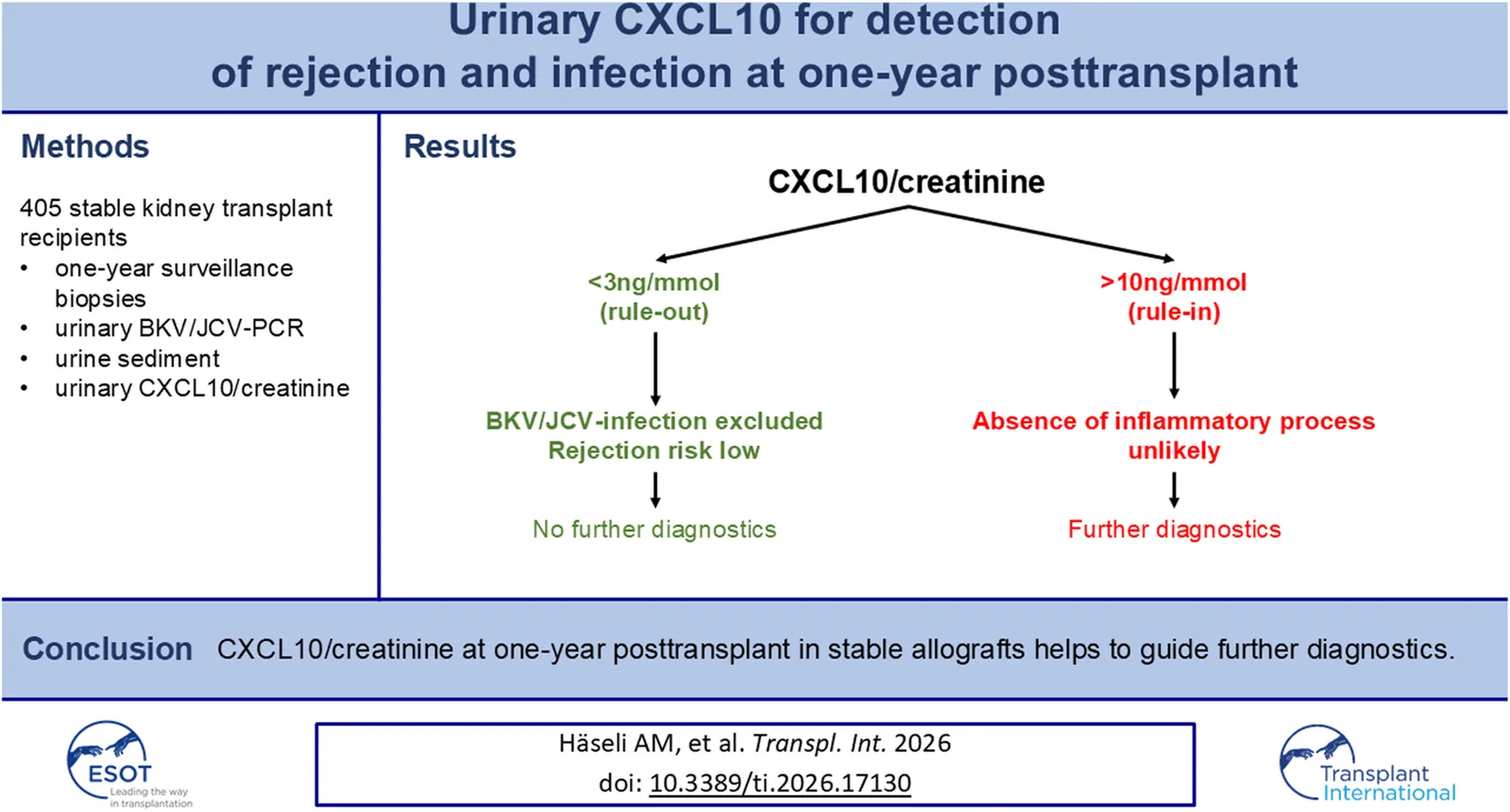

## Introduction

Urinary C-X-C motif chemokine ligand 10 (CXCL10) has been shown to correlate strongly with inflammatory processes in the renal allograft such as clinical/subclinical rejection, BK polyomavirus (BKV) infection, and urinary tract infection (UTI) [1–9]. Furthermore, ischemia-reperfusion injury, JC polyomavirus (JCV) infection, cytomegalovirus (CMV) infection, and other systemic infections might lead to elevated urinary CXCL10 levels [7, 10, 11].

Previous studies investigated the diagnostic characteristics of urinary CXCL10 at various timepoints posttransplant and with/without allograft dysfunction [2, 7, 12, 13]. As the inflammatory processes leading to elevated CXCL10 levels change over time and their intensity might be different at later stages posttransplant, the diagnostic properties should ideally be investigated for dedicated timepoints posttransplant. There are currently very few data assessing the diagnostic utility of CXCL10 specifically in stable transplants 1-year posttransplant, which might also be informative for long-term monitoring beyond the first year [7].

Some groups used multivariable models including urinary CXCL9/CXCL10, BKV-DNAemia, allograft function (i.e., estimated glomerular filtration rate [eGFR]), presence/absence of UTI, and presence/absence of *de novo* donor-specific antibodies (DSA), and patient age and sex to calculate the risk of rejection [7, 12, 14]. Such models can account for confounders but might be limited by their ease of use, because not all required data are available or current at the time of the investigation. It would be beneficial if a single measurement of urinary CXCL10 could inform on the inflammatory status of the allograft and help to guide further diagnostic steps.

The aim of this study was to investigate the diagnostic characteristics of urinary CXCL10 to detect major inflammatory processes in stable renal allograft recipients at 1-year posttransplant.

## Materials and methods

### Patient population and urine collection

The study was approved by the local ethics committee (EKNZ 2026-00071). This is a retrospective study based on all renal transplantations performed at the University Hospital Basel from January 2014 until August 2024. During this time frame, 825 transplantations were performed. All consenting adult kidney transplant recipients who had an informative 1-year surveillance biopsy and complete standard urine sample data were included (Figure 1). The standard urine analysis at the timepoint of the 1-year surveillance biopsy included a urine sediment and measurements of total proteinuria (protein/creatinine ratio; normal range <11 mg/mmol), albuminuria (albumin/creatinine ratio; normal range <3 mg/mmol), and α1-microglobulinuria (α1-microglobulin/creatinine ratio; normal range <1.58 mg/mmol). Aliquots of these urine samples were stored at −75 °C for later use.

**FIGURE 1:**
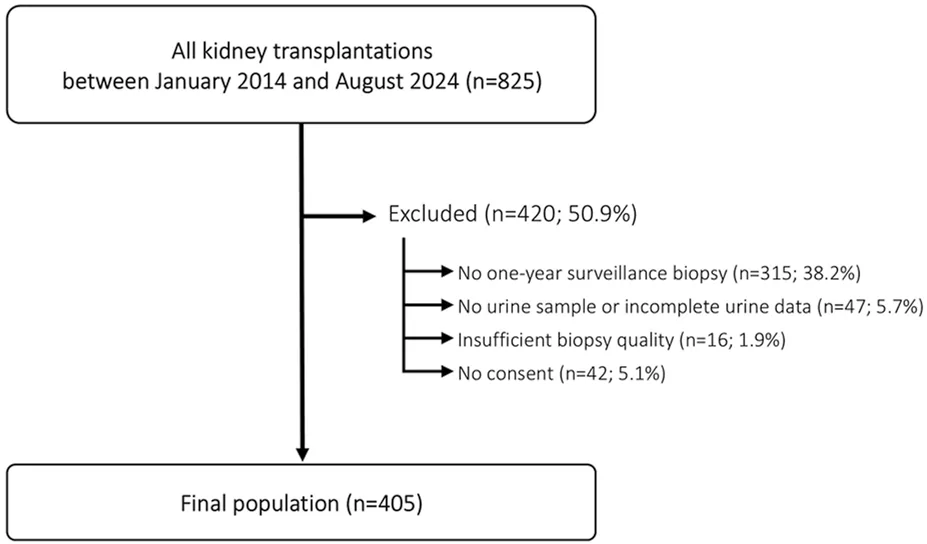
Patient flowchart.

### Urine analyses and definition of inflammatory processes

Urine CXCL10 was measured with the MSD V-Plex Human IP-10 Gen. B Kits (Meso Scale Discovery) and is given as CXCL10/creatinine ratios [ng/mmol creatinine] as previously reported [15]. Urine creatinine was measured by an enzymatic method on a Roche Cobas 8100 instrument. Urine cell components were analyzed by flowcytometry. BKPyV- and JCPyV-DNAuria were quantified retrospectively from stored aliquots using well-validated, species-specific laboratory-developed quantitative PCR assays as previously described [16–18]. Significant leukocyturia, relevant BKV- or JCV-DNAuria, and subclinical rejection were considered as individual inflammatory processes. If several such processes occurred simultaneously, they were summarized as “combination” of inflammatory processes. The individual inflammatory processes were defined as follows: (i) relevant DNAuria was defined as >10^6^ copies per milliliter [c/mL] for BKV and >10^7^ c/mL for JCV in accordance with the recently published international consensus guidelines [19]; (ii) significant leukocyturia was defined as >10 leukocytes per high power field (HPF), which corresponds in our laboratory to 56 leukocytes/μL; and (iii) rejection was defined by the Banff 2022 classification [20].

### Statistical analysis

If the measured values were below the detection threshold (e.g., protein/creatinine ratio), for analytical purposes we used a value of half the detection threshold instead. In addition, urinary leukocyte counts of <1/HPF were set as zero.

Categorical data are shown as count (percentage) and analyzed by Χ^2^-test. In the absence of a normal distribution, continuous data are presented as median (interquartile range). Receiver operating characteristics (ROC) were performed to assess the diagnostic characteristics of urinary CXCL10/creatinine ratio, serum creatinine, protein/creatinine ratio, albumin/creatinine ratio, and α1-microglobulin/creatinine ratio to detect inflammatory processes. A multivariable model was used to assess independently associated parameters with subclinical allograft rejection. It included all clinical and laboratory parameters that had a p-value <0.10 in the univariate analysis. The variable selection for the univariate analysis was based on established risk factors for allograft rejection as well as known confounders for urinary CXCL10/creatinine levels (i.e., relevant BKV-DNAuria, relevant JCV-DNAuria, and significant leukocyturia). A two-sided p-value of <0.05 was considered statistically significant. For statistical analysis we used IBM SPSS Statistics version 31.0.0.0 (117), JMP Pro 18, and R statistical software^1^.

## Results

### Population characteristics

In the study, 405 patients were included (Figure 1). The median recipient age was 55 years (44-63), and a third of all participants were female. Deceased donors accounted for 58% of the allografts and median donor age was 56 years (47-63). Twenty-two percent of the transplantations were considered as high-immunological risk (i.e., pretransplant donor-specific HLA-antibodies [HLA-DSA], ABO incompatibility, or husband-to-wife transplantation with mutual children). Induction therapy was mainly basiliximab (84%) and ATG (15%); the initial maintenance therapy was a triple therapy consisting of tacrolimus, mycophenolic-acid, and steroids in 99%. *De novo* HLA-DSA at 1-year posttransplant were detected in 11/405 patients (2.7%). The detailed population characteristics are summarized in Table 1.

**TABLE 1:** Population characteristics (n = 405).

Variable	Value
Recipient sex female	125 (30.9%)
Recipient age [years]	55 (44–63)
Underlying renal disease - ADPKD - Diabetic nephropathy - Glomerulonephritis/glomerulopathy - Interstitial nephropathy - Vascular nephropathy - Other nephropathies - Unknown nephropathy	86 (21.2%) 30 (7.4%) 148 (36.5%) 17 (4.2%) 39 (9.6%) 48 (11.9%) 37 (9.1%)
Deceased donor	235 (58.0%)
Donor age [years]	56 (47–63)
First transplant	358 (88.4%)
Immunological high-risk - AB0 incompatible - Pretransplant HLA-DSA - Other risk (husband-to-wife with mutual children) - Combinations (AB0 incompatible + pretransplant HLA-DSA/other risk)	90 (22.2%) 33 (8.1%) 43 (10.6%) 8 (2%) 6 (1.5%)
HLA-mismatches (A, B, C, DRB1, DRB3-5, DQB1, and DPB1)* (n = 398)	8 (6–10)
Induction therapy - ATG - Basiliximab - None	63 (15.6%) 341 (84.2%) 1 (0.2%)
Initial maintenance immunosuppression - Tac-MPA-P - Other	402 (99.3%) 3 (0.7%)
Delayed graft function	87 (21.5%)
Laboratory values at 1-year surveillance biopsy - Urine CXCL10/creatinine [ng/mmol] - Serum creatinine [µmol/L] - eGFR [mL/min] - Urine protein/creatinine ratio [mg/mmol] - Urine albumin/creatinine ratio [mg/mmol] - Urine α1-microglobulin/creatinine ratio [mg/mmol] - C-reactive protein [mg/L] - *De novo* HLA-DSA	2.41 (1.19–5.01) 125 (100–151) 56 (44–70) 10.10 (6.00–17.70) 2.02 (1.09–5.55) 3.38 (1.91–5.77) 1.50 (0.63–3.5) 11 (2.7%)
One-year surveillance biopsy - No rejection - T cell-mediated rejection - Antibody-mediated rejection - Mixed rejection	341 (84%) 23 (6%) 36 (9%) 5 (1%)

ADPKD: autosomal dominant polycystic kidney disease; HLA-DSA: donor-specific HLA, antibodies; ATG: anti-T-cell globulin; Tac: Tacrolimus; MPA: mycophenolic acid; P: prednisone. *High-resolution HLA-typing data not available for all patients (number with available data in brackets). Mismatches were calculated at the serological level.

### Inflammatory processes and their overlap

Two hundred and eighty-one of the 405 patients (69%) had no inflammatory process at the 1-year surveillance biopsy. The most frequent individual inflammatory process was rejection with 15.8% (n = 64), followed by leukocyturia with 11.6% (n = 47), BKV-DNAuria with 5.7% (n = 23), and JCV-DNAuria with 2.2% (n = 9). Eighteen patients (4.4%) showed an overlap of inflammatory processes. The most frequent combinations were leukocyturia with rejection in 12 patients and three patients with BKV-DNAuria and rejection. The distribution of individual inflammatory processes and their overlaps are shown in Figure 2A.

**FIGURE 2:**
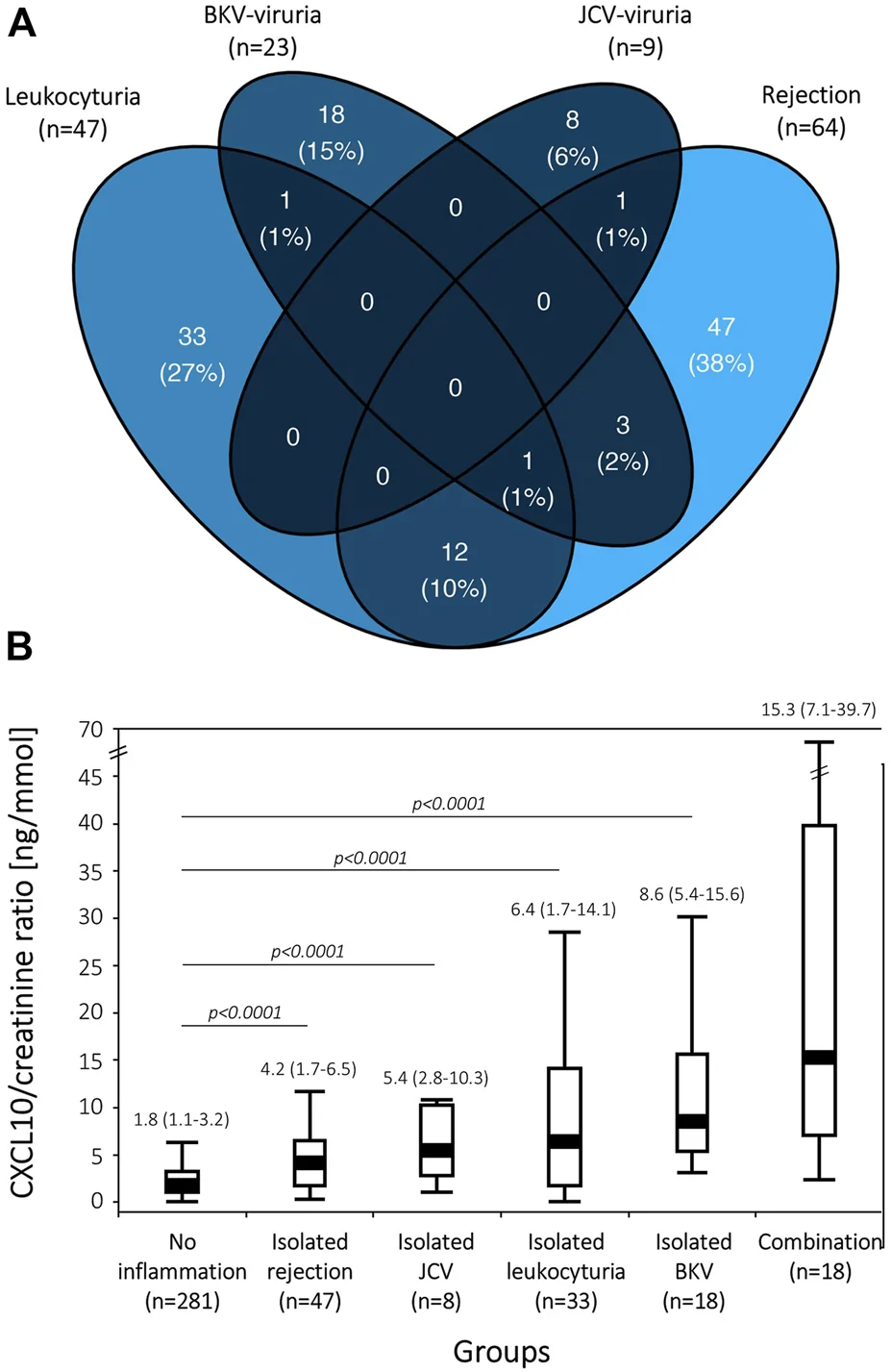
Overview of inflammatory processes. **(A)** Venn diagram illustrating the number of inflammatory processes and their overlap. **(B)** Urine CXCL10/creatinine ratio in different inflammatory processes in ascending order (median and interquartile ranges are given above the boxplots).

The 64 rejection phenotypes were classified as follows in descending order of frequency: borderline changes (n = 15, 3.7%), microvascular inflammation (MVI) without HLA-DSA and negative C4d (n = 12, 3.0%), active antibody-mediated rejection (AMR) (n = 11, 2.7%), chronic active AMR (n = 8, 2.0%), acute T-cell mediated rejection (TCMR) ≥ Banff IA (n = 7, 1.7%), C4d staining without evidence of rejection (n = 5, 1.2%), mixed rejection (n = 5, 1.2%), and chronic active TCMR (n = 1, 0.2%).

### Urinary CXCL10/creatinine levels in different inflammatory processes

The lowest urinary CXCL10/creatinine levels were observed in patients without any inflammatory processes (median CXCL10/creatinine 1.8 ng/mmol [1.1–3.2]). Urinary CXCL10/creatinine levels were significantly higher in all inflammatory processes compared to the group without any inflammation (all p < 0.0001; Figure 2B). The highest values were observed in patients with a combination of inflammatory processes (median CXCL10/creatinine 15.3 ng/mmol [7.1–39.7]). Interestingly, patients with isolated subclinical rejection had the lowest CXCL10/creatinine among those with any individual inflammatory processes (median CXCL10/creatinine 4.2 ng/mmol [1.7–6.5]). No correlation was found between C reactive protein (CRP) and urinary CXCL10/creatinine levels (r^2^ < 0.001, p = 0.91; data not shown). CXCL10/creatinine levels were not different between the eleven patients with *de novo* HLA-DSA at 1-year posttransplant and those without (3.6 ng/mmol [1.1–4.6] vs. 2.4 ng/mmol [1.2–5.0]; p = 0.83).

### Diagnostic characteristics of urinary CXCL10/creatinine

Of the cohort, 124 patients (31%) had at least one inflammation process. The ROC-analysis revealed an AUC of 0.786 (95%-CI: 0.727–0.836) for CXCL10/creatinine (p < 0.0001) to detect any inflammation process. In contrast, none of the standard laboratory parameters had a statistically significant AUC: 0.499 (95%-CI: 0.436–0.562) for serum creatinine (p = 0.68), 0.561 (95%-CI: 0.498–0.621) for eGFR (p = 0.07), 0.636 (95%-CI: 0.575–0.692) for protein/creatinine (p = 0.05), 0.615 (95%-CI: 0.555–0.672) for albumin/creatinine (p = 0.18), and 0.553 (95%-CI: 0.491–0.613) for α1-microglobulin/creatinine (p = 0.26). The AUC for CXCL10/creatinine was significantly higher than for all other parameters (all p < 0.0001; Figure 3A). Next, we performed the same analyses for specific subgroups. The results were almost identical, if 47 cases with leukocyturia were excluded (AUC = 0.783 [95%-CI: 0.713–0.840]; Figure 3B). For detection of isolated relevant BKV-DNAuria (n = 18), the AUC was 0.936 (95%-CI: 0.886–0.965) and significantly higher than all other parameters (all p < 0.0001; Figure 3C). For detection of isolated subclinical rejection (n = 47), the AUC was lower at 0.703 (95%-CI: 0.607–0.783) but still significantly higher than all other parameters (all p < 0.006; Figure 3D). The AUC for isolated relevant JCV-DNAuria (n = 8) and isolated significant leukocyturia (n = 33) were 0.808 (95%-CI: 0.585–0.926) and 0.736 (95%-CI: 0.597–0.840), respectively (data not shown). In a subgroup analysis of immunological standard-risk (n = 315) and high-risk patients (n = 90), we found almost identical results for the standard-risk group (AUC = 0.804 [95%-CI: 0.736–0.857]; p < 0.0001 vs. all other parameters), while the AUC was lower in the high-risk group (AUC = 0.737 [95%-CI: 0.604–0.837]; p < 0.05 vs. all parameters except protein/creatinine ratio) (Supplementary Figure 1).

**FIGURE 3:**
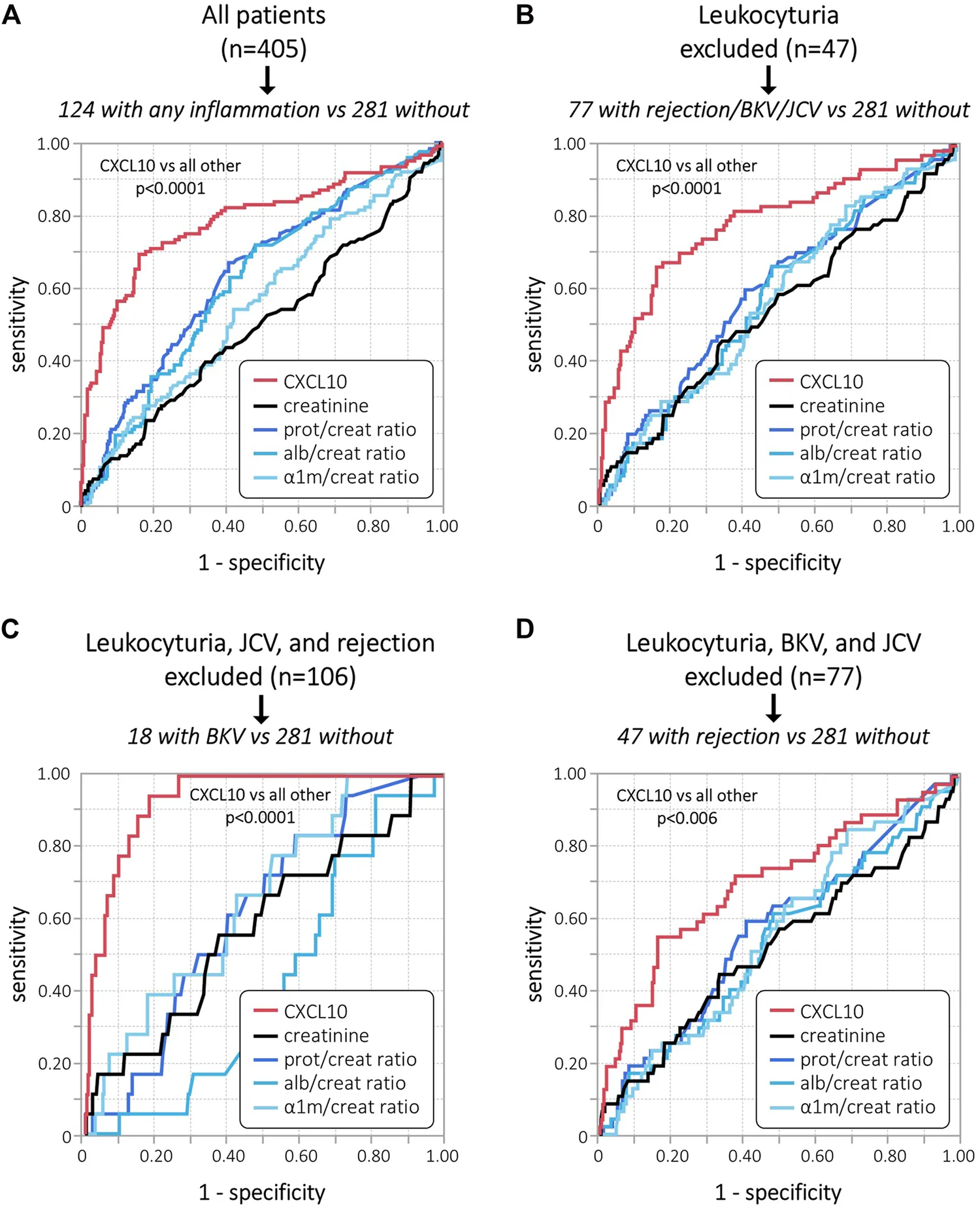
ROC analyses. **(A)** All patients (AUC = 0.786; 95%-CI: 0.727–0.836). **(B)** Patients with significant leukocyturia excluded (AUC = 0.783; 95%-CI: 0.713–0.840). **(C)** Patients with isolated relevant BKV-DNAuria (AUC = 0.936; 95%-CI: 0.886–0.965). **(D)** Patients with isolated subclinical rejection (AUC = 0.703; 95%-CI: 0.607–0.783).

### Determination of preliminary rule-out and rule-in cutoff

The details of the diagnostic characteristics of urinary CXCL10/creatinine to detect inflammatory processes at various cutoff levels are summarized in Table 2. The optimal cutoff according to the Youden-index was 3.97 ng/mmol with a sensitivity of 69% and a specificity of 84%. The negative likelihood ratio (LR-) was 0.36, the positive likelihood ratio (LR+) was 4.34. Next, we defined two arbitrary cutoffs to rule-in or rule-out inflammatory processes in an efficient way. At a low rule-out cutoff of 3 ng/mmol with a LR- of 0.36, 51% of uninformative surveillance biopsies could be avoided while maintaining a sensitivity of 73% for detecting inflammatory processes. Importantly, 33/124 (27%) inflammatory processes would be missed, among which subclinical rejection was the most frequent (n = 20). The most commonly missed rejection phenotypes were borderline changes (n = 5) and various forms of AMR/MVI (see footnotes in Table 2). No relevant BKV-DNAuria ere missed, and only 2/9 cases with relevant JCV-DNAuria were missed. At a high rule-in cutoff of 10 ng/mmol with a LR+ of 12.92, only 2% had no inflammatory process. The same analyses were also performed excluding patients with relevant leukocyturia (n = 47) and are summarized in Table 3. The results were very similar to the whole cohort.

**TABLE 2:** Diagnostic characteristics including all patients (n = 405). Of the 405 patients, 124 had at least one inflammatory process (31%). The AUC in the ROC-analysis was 0.786 (95%-CI: 0.727–0.836).

CXCL10/Cr Cutoff [ng/mmol]	Sens Spec	PPV NPV	LR+ LR-	Absence of any inflammation above cutoff – false positive (n/281)	Correctly avoided biopsies – true negative (n/405)	Missed inflammatory processes below cutoff – false negative (n/124)
1	92% 22%	34% 86%	1.17 0.37	220/281 (78%)	61/405 (15%)	10/124 (8%) Leukocyturia (n = 5), BKV (n = 0), JCV (n = 0), rejection (n = 5)^#^, combo (n = 0)
2	82% 57%	46% 88%	1.90 0.31	122/281 (43%)	159/405 (39%)	22/124 (18%) Leukocyturia (n = 8), BKV (n = 0), JCV (n = 1), rejection (n = 13)^##^, combo (n = 0)
3	73% 73%	54% 86%	2.72 0.36	76/281 (27%)	205/405 (51%)	33/124 (27%) Leukocyturia (n = 9), BKV (n = 0), JCV (n = 2), rejection (n = 20)^###^, combo (n = 2)
3.97*	69% 84%	66% 86%	4.34 0.36	45/281 (16%)	236/405 (58%)	38/124 (31%) Leukocyturia (n = 10), BKV (n = 2), JCV (n = 3), rejection (n = 21)^####^, combo (n = 2)
6	49% 93%	74% 81%	6.56 0.55	21/281 (7%)	260/405 (64%)	63/124 (51%) Leukocyturia (n = 16), BKV (n = 6), JCV (n = 5), rejection (n = 33)^#####^, combo (n = 3)
10	33% 98%	85% 77%	12.92 0.69	7/281 (2%)	274/405 (68%)	84/124 (68%) Leukocyturia (n = 22), BKV (n = 10), JCV (n = 6), rejection (n = 38)^######^, combo (n = 8)

Sens: Sensitivity; Spec: Specificity; PPV: positive predictive value; NPV: negative predictive value; LR+: positive likelihood ratio; LR−: negative likelihood ratio; BKV: BK, polyomavirus; JCV: JC, polyomavirus; combo: combination of inflammatory processes; Cr: creatinine.

* Threshold with highest Youden-Index.

^#^C4d staining without evidence of rejection (n = 2), Chronic active AMR (n = 2), MVI, without HLA-DSA, and C4d negative (n = 1).

^##^Active AMR (n = 2), Borderline changes (n = 4), C4d staining without evidence of rejection (n = 2), Chronic active AMR (n = 3), Mixed rejection (n = 1), MVI, without HLA-DSA, and C4d negative (n = 1).

^###^Active AMR (n = 3), Borderline changes (n = 5), C4d staining without evidence of rejection (n = 3), Chronic active AMR (n = 3), Chronic active TCMR (n = 1), Mixed rejection (n = 2), MVI, without HLA-DSA, and C4d negative (n = 3).

^####^Active AMR (n = 4), Borderline changes (n = 5), C4d staining without evidence of rejection (n = 3), Chronic active AMR (n = 3), Chronic active TCMR (n = 1), Mixed rejection (n = 2), MVI, without HLA-DSA, and C4d negative (n = 3).

^#####^Active AMR (n = 5), Acute TCMR (n = 1) Borderline changes (n = 9), C4d staining without evidence of rejection (n = 4), Chronic active AMR (n = 5), Chronic active TCMR (n = 1), Mixed rejection (n = 2), MVI, without HLA-DSA, and C4d negative (n = 6).

^######^Active AMR (n = 5), Acute TCMR (n = 3) Borderline changes (n = 10), C4d staining without evidence of rejection (n = 4), Chronic active AMR (n = 6), Chronic active TCMR (n = 1), Mixed rejection (n = 2), MVI, without HLA-DSA, and C4d negative (n = 7).

**TABLE 3:** Diagnostic characteristics excluding 47 patients with leukocyturia, leaving 358 patients for the analysis. Seventy-seven of 358 patients had at least one inflammatory process (22%). The AUC in the ROC-analysis was 0.783 (95%-CI: 0.713–0.840).

CXCL10/Cr Cutoff [ng/mmol]	Sens Spec	PPV NPV	LR+ LR-	Absence of any inflammation above cutoff – false positive (n/281)	Correctly avoided biopsies – true negative (n/358)	Missed inflammatory processes below cutoff – false negative (n/77)
1	94% 22%	25% 92%	1.19 0.30	220/281 (78%)	61/358 (17%)	5/77 (6%) BKV (n = 0), JCV (n = 0), rejection (n = 5)^#^, combo (n = 0)
2	82% 57%	34% 92%	1.89 0.32	121/281 (43%)	160/358 (45%)	14/77 (18%) BKV (n = 0), JCV (n = 1), rejection (n = 13)^##^, combo (n = 0)
3	71% 73%	42% 90%	2.64 0.39	76/281 (27%)	205/358 (57%)	22/77 (29%) BKV (n = 0), JCV (n = 2), rejection (n = 20)^###^, combo (n = 0)
3.97*	66% 84%	53% 90%	4.14 0.40	45/281 (16%)	236/358 (66%)	26/77 (34%) BKV (n = 2), JCV (n = 3), rejection (n = 21)^####^, combo (n = 0)
6	43% 93%	61% 86%	5.72 0.62	21/281 (7%)	260/358 (73%)	44/77 (57%) BKV (n = 6), JCV (n = 5), rejection (n = 33)^#####^, combo (n = 0)
10	29% 98%	76% 83%	11.47 0.73	7/281 (2%)	274/358 (77%)	55/77 (71%) BKV (n = 10), JCV (n = 6), rejection (n = 38)^######^, combo (n = 1)

Sens: Sensitivity; Spec: Specificity; PPV: positive predictive value; NPV: negative predictive value; LR+: positive likelihood ratio; LR-: negative likelihood ratio; BKV: BK, polyomavirus; JCV: JC, polyomavirus; combo: combination of inflammatory processes; Cr: creatinine.

* Threshold with highest Youden-Index.

^#^ C4d staining without evidence of rejection (n = 2), Chronic active AMR (n = 2), MVI, without HLA-DSA, and C4d negative (n = 1).

^##^Active AMR (n = 2), Borderline changes (n = 4), C4d staining without evidence of rejection (n = 2), Chronic active AMR (n = 3), Mixed rejection (n = 1), MVI, without HLA-DSA, and C4d negative (n = 1).

^###^Active AMR (n = 3), Borderline changes (n = 5), C4d staining without evidence of rejection (n = 3), Chronic active AMR (n = 3), Chronic active TCMR (n = 1), Mixed rejection (n = 2), MVI, without HLA-DSA, and C4d negative (n = 3).

^####^Active AMR (n = 4), Borderline changes (n = 5), C4d staining without evidence of rejection (n = 3), Chronic active AMR (n = 3), Chronic active TCMR (n = 1), Mixed rejection (n = 2), MVI, without HLA-DSA, and C4d negative (n = 3).

^#####^Active AMR (n = 5), Acute TCMR (n = 1) Borderline changes (n = 9), C4d staining without evidence of rejection (n = 4), Chronic active AMR (n = 5), Chronic active TCMR (n = 1), Mixed rejection (n = 2), MVI, without HLA-DSA, and C4d negative (n = 6).

^######^Active AMR (n = 5), Acute TCMR (n = 3) Borderline changes (n = 10), C4d staining without evidence of rejection (n = 4), Chronic active AMR (n = 6), Chronic active TCMR (n = 1), Mixed rejection (n = 2), MVI, without HLA-DSA, and C4d negative (n = 7).

### Multivariable analysis

Finally, we investigated independently associated parameters with subclinical rejection in a multivariable model. Urinary CXCL10/creatinine, proteinuria, eGFR, patient age, immunological high-risk (i.e., pretransplant HLA-DSA and/or ABO incompatible transplant), leukocyturia, and *de novo* HLA-DSA had p-values <0.10 in the univariate analysis. In the multivariable analysis, only urinary CXCL10/creatinine (odds ratio 1.03 [95% CI: 1.01–1.05]; p = 0.0008) and immunological high-risk (odds ratio 2.14 [95% CI: 1.16–3.96]; p = 0.02) were independently associated with subclinical rejection (Table 4). Forcing relevant BKV-DNAuria and JCV-DNAuria as known confounders for urinary CXCL10/creatinine levels into the multivariate model did not change the results. Urinary CXCL10/creatinine (odds ratio 1.03 [95% CI: 1.01–1.06]; p = 0.0006) and immunological high-risk (odds ratio 2.14 [95% CI: 1.16–3.97]; p = 0.02) were still the only significantly associated parameters with subclinical rejection, while relevant BKV-DNAuria (odds ratio 0.58 [95% CI: 0.15–2.28]; p = 0.41) and JCV-DNAuria (odds ratio 0.80 [95% CI: 0.10–6.69]; p = 0.83) were not.

**TABLE 4:** Univariate and multivariable analysis for allograft rejection. Sixty-four of 405 patients (15.8%) had rejections in the 1-year surveillance biopsy according to the Banff 2022 classification.

​	Univariate analysis			Multivariable analysis	
Parameter	Rejection (n = 64)	No rejection (n = 341)	p-value	Odds ratio (95% CI)	p-value
Urine CXCL10/creatinine [ng/mmol]	4.92 (2.42–13.15)	2.05 (1.11–4.37)	<0.0001	1.03 (1.01–1.05)	0.0008
Urine protein/creatinine [mg/mmol]	13.2 (7.9–32.9)	9.9 (6.0–16.3)	0.002	1.01 (0.99–1.01)	0.19
eGFR [mL/min]	51 (42-62)	57 (45-70)	0.06	0.99 (0.98–1.01)	0.47
Patient age	58 (50-64)	53 (44-62)	0.01	1.02 (0.99–1.05)	0.12
Total HLA-mismatches*	8 (6-10)	8 (6-10)	0.27	​	​
Immunological high-risk (n = 90)**	Yes: 23% No: 14%	Yes: 77% No: 86%	0.03	2.14 (1.16–3.96)	0.02
Relevant BKV-DNAuria (n = 23)	Yes: 17% No: 16%	Yes: 83% No: 84%	0.83	​	​
Relevant JCV-DNAuria (n = 9)	Yes: 11% No: 16%	Yes: 89% No: 84%	0.70	​	​
Female sex (n = 125)	Yes: 18% No: 15%	Yes: 82% No: 85%	0.34	​	​
Deceased donor (n = 235)	Yes: 17% No: 14%	Yes: 83% No: 86%	0.43	​	​
*De novo* HLA-DSA (n = 11)	Yes: 36% No: 15%	Yes: 64% No: 85%	0.06	3.51 (0.92–13.43)	0.08
Leukocyturia >10/HPF (n = 47)	Yes: 28% No: 14%	Yes: 72% No: 86%	0.02	1.34 (0.58–3.07)	0.50
Prior renal transplantation (n = 47)	Yes: 17% Ne: 16%	Yes: 83% No: 84%	0.81	​	​

* Includes HLA-loci A/B/C/DRB1/DRB3-5/DQB1/DPB1.

** Pretransplant HLA-DSA, and/or ABO-incompatible transplant and/or husband-to-wife with mutual children.

## Discussion

The key finding of this study was that a single urinary CXCL10/creatinine measurement in stable renal allograft recipients at 1-year posttransplant offers the potential to rule-in/rule-out inflammatory processes and guide further diagnostics. With a urinary CXCL10/creatinine value <3 ng/mmol (rule-out; 59% of all patients), relevant BKV-DNAuria can be excluded, the risk of relevant JCV-DNAuria is low (2/405; 0.5%), and the risk of subclinical rejection drops from 15.8% to 5.4%. In addition, with a urinary CXCL10/creatinine value > 10 ng/mmol (rule-in), the absence of any inflammatory process is very unlikely, prompting further diagnostics.

The lowest AUC of urinary CXCL10/creatinine was observed for detection of subclinical rejection compared to viral or bacterial infections. While measurement of leukocyturia is routinely performed at all posttransplant follow-up visits, most centers have a less dense screening schedule to assess BKV replication, and JCV replication is rarely measured. Our study suggests that the incremental value of urinary CXCL10/creatinine in routine practice has potential to detect all inflammatory processes in one single assay, allowing further diagnostic steps.

It is important to note that the proposed rule-out and rule-in cutoffs are arbitrary. According to Tables 2 and 3, the rule-out cutoff could be set lower, which will reduce the number of missed subclinical rejections even further. Centers performing surveillance biopsies routinely at 1-year posttransplant might consider the missed subclinical rejection rate of 5.4% as still too high, while centers not performing surveillance biopsies might find this false negative rate as acceptable because most subclinical rejection episodes would be detected.

Our data are overall in line with previous publications indicating the diagnostic value of urinary CXCL10/creatinine [3, 4, 6, 7, 9, 12, 21–25]. Some groups used urinary CXCL10/creatinine together with CXCL9/creatinine and several other parameters (i.e., eGFR, presence of HLA-DSA, BKV-DNAemia) in a data model to improve prediction of rejection [7, 14]. While this approach is elegant, the models were developed in a mixture of clinical and subclinical rejection episodes at various timepoints posttransplant. Therefore, the reported prediction model of rejection might not be fully appropriate for stable transplants 1-year posttransplant and beyond.

Recently, Goutaudier et al. found, in a large multicenter cohort, no additional diagnostic value of urinary CXCL10/creatinine to predict clinical/subclinical rejection compared to a set of clinical standard-of-care parameters (i.e., prior transplantation, donor type, history of rejection, delayed graft function, HLA-A/B/DR/DQ mismatches, induction with thymoglobulin, eGFR, HLA-DSA, and BKV-DNAemia) [13]. In our cohort, only urinary CXCL10/creatinine and immunological high-risk status were independently associated with subclinical rejection at 1-year posttransplant. We can only speculate on this discrepancy, which might be related to differences in the cohorts, the definition of rejection, and the time frame of analyses. Indeed, Goutadier et al. used the Banff 2019 classification considering borderline/suspicious for acute TCMR, suspicious for AMR, and chronic inactive AMR not as rejection. However, these rejection phenotypes accounted for almost half of all detected rejection episodes in their cohort. Furthermore, the standard-of-case model was built on both clinical and surveillance biopsies, and 41% of all included rejection episodes were detected in clinical biopsies. This could explain why, for example, eGFR was predictive for rejection in their model but not in our cohort consisting of only surveillance biopsies in stable transplants at 1-year posttransplant.

Interestingly, Van Loon et al. found an association between urinary CXCL10/creatinine and CMV viremia as well as CRP [7]. We did not observe a correlation between urinary CXCL10 and CRP, but the association with CMV viremia could not be assessed, because it was not measured systematically at 1-year posttransplant. We acknowledge that CMV infection and major systemic infections reflected by high CRP might increase urinary CXCL10/creatinine levels. However, these confounders are rare in stable transplant recipients at 1-year posttransplant.

While a CXCL10/creatinine <3 ng/mmol (rule-out) almost excludes any relevant bacterial and viral urinary tract infection, around a third of subclinical rejection processes are missed. Histology is still considered the gold standard for diagnosis of rejection. This has been challenged by new diagnostic platforms like the molecular microscope diagnostic system (MMDx), highlighting that processes can be differently captured by conventional histology and on a molecular level [26, 27]. We currently consider those subclinical rejection episodes with low urinary CXCL10/creatinine <3 ng/mmol as false negative CXCL10 tests. Alternatively, these rejection episodes with low urinary CXCL10/creatinine levels could have limited inflammatory activity and might not be associated with induction of significant damage. This hypothesis can only be tested in a prospective trial, in which rejection episodes with low CXCL10/creatinine are either treated or left untreated.

Intriguingly, the diagnostic performance of urinary CXCL10/creatinine was worse in immunological high-risk patients (n = 90). This could either point to a statistical power problem or a lower inflammation grade in these rejection episodes due to a higher overall immunosuppression load (i.e., induction with ATG and long-term triple immunosuppression). Further studies are required in immunological high-risk patients, and they might benefit from standard surveillance biopsies, even if urinary CXCL10/creatinine is low.

Urinary CXCL10/creatinine is a nonspecific inflammation biomarker, while donor-derived cell-free DNA (dd cfDNA) in blood is a nonspecific injury biomarker, which has recently been studied for detection of renal allograft rejection [28–31]. Urinary CXCL10/creatinine has some advantages compared to dd cfDNA: (i) low levels do not only indicate a low risk of rejection but can also exclude relevant BKV-DNAuria with high certainty; (ii) simplicity of the assay; (iii) fast turn-around-time; and (iv) low costs [8, 14, 15, 32]. To the best of our knowledge, a direct comparison of urinary CXCL10/creatinine and dd cfDNA has so far only been performed in small case-control studies [33–35]. Overall, the current data suggest that dd cfDNA detects AMR processes better than urinary CXCL10/creatinine, while the latter performs better regarding detection of TCMR, especially tubulointerstitial rejection [35]. Therefore, the two biomarkers might supplement each other as they reflect different rejection phenotypes in different compartments, but this should be investigated in larger unselected cohorts or prospective interventional studies. Indeed, a panel of different biomarkers might improve the diagnostic accuracy, but this must be balanced against the increased complexity and costs [7, 14].

The advantages of this study are the reasonably large population size, the broad inclusion criteria reflecting real-life, and its restriction to a specific timepoint posttransplant leading to a robust assessment of the diagnostic characteristics. In addition, we measured BKV-DNAuria and JCV-DNAuria in all patients, which was not done in other studies.

This study has some limitations. First, it is a single-center study on predominantly Caucasian patients, which may limit generalizability to other kidney transplant programs with different patient populations or different immunosuppressive regimens. Second, we have no external validation cohort. Although the diagnostic robustness of urinary CXCL10/creatinine alone has been proven in many cohorts, validation of the specific CXCL10/creatinine cutoffs and the proposed diagnostic algorithm is required, especially if another analytical platform is used (e.g., Luminex- or ELLA-based CXCL10 measurement systems) or different clinical settings are investigated. Third, we are unable to include dd cfDNA measurements in this study because no plasma samples were collected in the majority of the 405 patients.

In conclusion, this study supports urinary CXCL10/creatinine measurements at 1-year posttransplant in stable allografts to detect inflammatory processes and guide further diagnostics. The preliminary cutoffs require external validation.

## Data Availability

The raw data supporting the conclusions of this article will be made available by the authors, without undue reservation.

## Footnotes

1
www.r-project.org
