# Supplementary material for: Urinary CXCL10 for detection of rejection and infection at 1-year posttransplant
Source: Transpl Int. 2026 Oct 12;39:17130. doi: 10.3389/ti.2026.17130 (PMC13696364; doi:10.3389/ti.2026.17130)

Capsule Sentence Summary

This study suggests that urinary CXCL10 can help to guide further diagnostic steps (e.g. allograft biopsy) in stable patients one-year after kidney transplantation. It outperforms standard diagnostic parameters such as serum creatinine and proteinuria.

Supplementary Figure legends

Supplementary Figure 1. ROC-Analysis. **A**: All standard-risk patients (AUC=0.804; 95%-CI: 0.736-0.857). **B**: All high-risk patients (AUC=0.737; 95%-CI: 0.604-0.837). **C**: All standard-risk patients with significant leukocyturia excluded (AUC=0.804; 95%-CI: 0.726-0.865). **D**: All high-risk patients with significant leukocyturia excluded (AUC=0.727; 95%-CI: 0.563-0.847).

Supplementary Figure 1

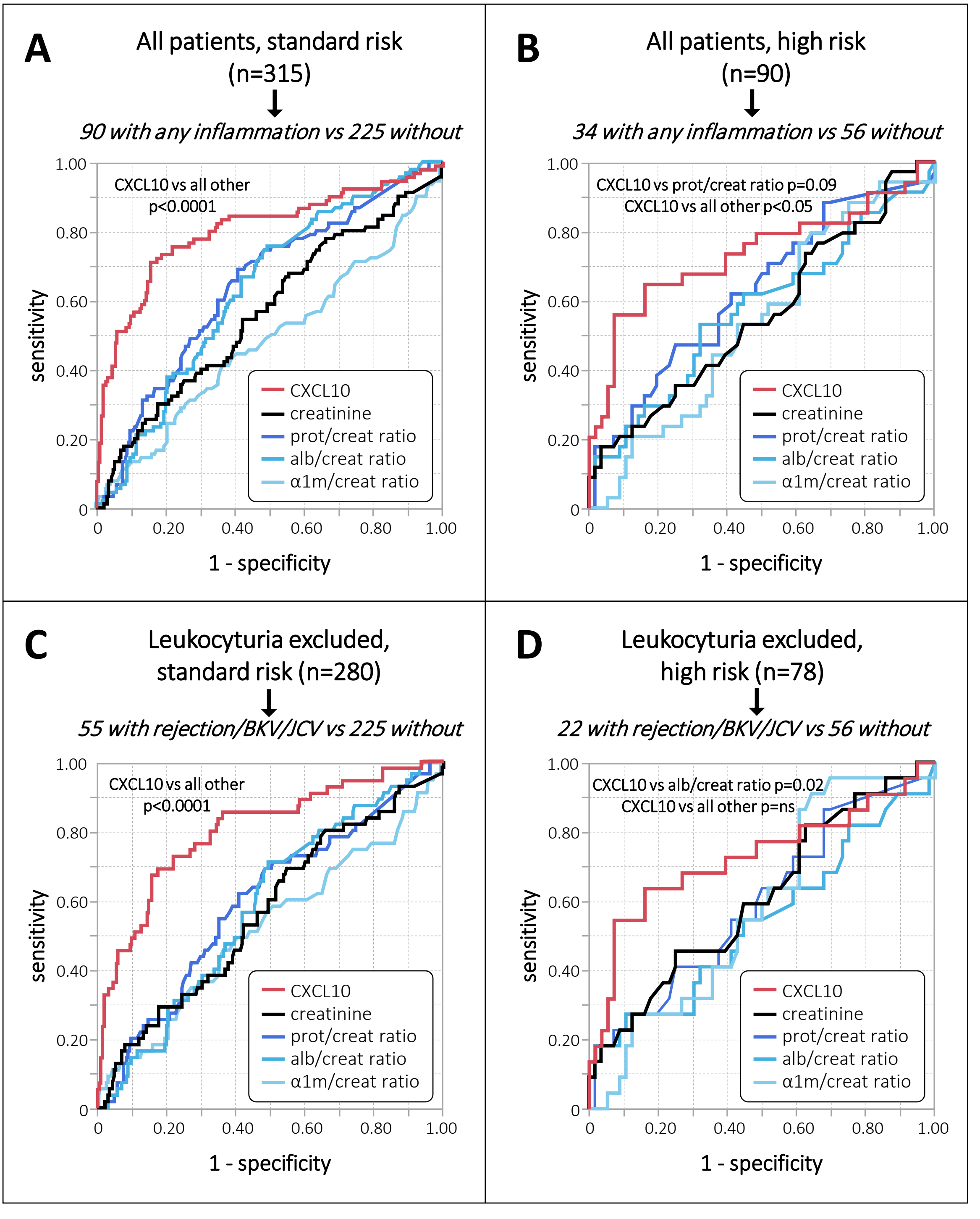

Supplement: Supplementary file 1 [file DataSheet1.pdf]
